# Visual Analysis of Panoramic Radiographs among Pediatric Dental Residents Using Eye-Tracking Technology: A Cross-Sectional Study

**DOI:** 10.3390/children10091476

**Published:** 2023-08-29

**Authors:** Ghalia Y. Bhadila, Safiya I. Alsharif, Seba Almarei, Jamila A. Almashaikhi, Dania Bahdila

**Affiliations:** 1 Department of Pediatric Dentistry, King Abdulaziz University, Jeddah 21589, Saudi Arabia; jamila.almashaikhi@gmail.com (J.A.A.); dbahdila@kau.edu.sa (D.B.); 2 General Dentist, King Abdulaziz University, Jeddah 21589, Saudi Arabia; safiyaalsharif7@gmail.com (S.I.A.); sebaalmarei@gmail.com (S.A.)

**Keywords:** eye tracking technology, gaze tracking, jaw cysts, pediatric dentistry, panoramic radiography

## Abstract

The aim of this cross-sectional study was to explore the eye tracking (ET) performance of postgraduate pediatric dental students in correctly detecting abnormalities in different sets of panoramic radiographs. This observational study recruited postgraduate pediatric dental students to evaluate seven panoramic radiographs. RED-m^®^ SMI software (Sensomotoric Instruments, Teltow, Germany) was used to track the participants’ eye movements as they looked at the radiographs. The data collected for areas of interest (AOIs) included revisit counts, fixation counts, fixation times, entry times, and dwell times. Univariate and bivariate analyses were conducted to summarize the participants’ characteristics and ET measures. The overall percentage of correctly located AOIs was 71.7%. The residents had significantly more revisits and fixation counts in AOIs located in one sextant than in multiple sextants (*p* < 0.001). Similar patterns were observed for fixation and dwell times (*p* < 0.001), but not for entry time. Heatmaps showed that the highest density of fixations was on the AOIs and the residents fixated more on dentition than on bony structures. In single-sextant radiographs, residents had significantly more revisits and fixation counts for AOIs compared to those of multiple sextants. Residents had slower entry times and dwelled less on AOIs located in multiple sextant(s). The reported findings can direct dental educators to develop a standardized scan scheme of panoramic radiographs to minimize misdiagnosis.

## 1. Introduction

Dental students’ competency in recognizing anatomical landmarks, identifying errors, and detecting artifacts in panoramic radiographs was reported [1,2]. However, knowledge of the average percentage of accurate responses to questions related to the correct interpretation of radiographic images is insufficient. Objectively showcasing the mechanism of the interpretation process that can considerably affect the diagnosis is of value. Despite the growing body of research on eye tracking technology (ETT) in dentistry [3,4,5], the mechanism of the interpretation process using ETT in the dental field has not been fully explored.

Several studies have reported the use of ETT to understand how clinicians use visual searches for their diagnostic decisions [6]. Recently, ETT has been used for interpreting medical images, including brain magnetic resonance imaging (MRI) [7], neuropsychological research [8], and many other fields [9,10,11]. ETT has been implemented to capture the subject’s gaze positions and patterns objectively and analyze these data via mathematical algorithms to develop reliable measures [12]. It can uncover various learning opportunities for understanding and analyzing information, which involves differences between novices and experts in decision-making and the process behind misdiagnosis and misinterpretation in both the dental and medical fields. Although the use of ETT has gained popularity in dental research [6], no studies have investigated its utilization in radiographic identification of pediatric jaw lesions in dental panoramic images among pediatric dental residents. 

Pediatric jaw lesions are often asymptomatic and discovered incidentally by dental practitioners on routine radiographic examinations [13]. They can be benign or malignant, and can occur around the tooth’s root, crown, or interradicular area, or can be unrelated to the teeth [14]. Pediatric tooth and jaw anomalies are common, which emphasizes the value of panoramic radiographs in detecting these lesions [15]. The use of radiological dental imaging is a valuable diagnostic tool when used in conjunction with clinical examination to diagnose pathological conditions among children [16]. Misinterpretations and missed lesion detection can lead to delayed diagnosis and subsequent patient care [17].

Panoramic radiographs can be challenging to interpret due to the wide coverage of anatomy in addition to superimpositions and ghost images. This can be particularly challenging for students who are still familiarizing themselves with the complex anatomy of the oral cavity, which poses a significant challenge in terms of interpretation [18]. Additionally, the presence of artifacts in panoramic radiographs can complicate the process of reading them, especially for junior dental students. These artifacts can obscure or distort important structures. Students must learn to differentiate between true anatomical variations and artifacts that may mimic abnormalities [19]. To overcome these challenges, dental students should strive to enhance their knowledge of dental anatomy and radiographic interpretation, which can potentially be achieved by new technologies, such as the use of ETT.

Studies examining the efficacy of ETT in the detection of pediatric jaw lesions in panoramic images among pediatric dental residents are lacking. Therefore, this study aimed to explore the eye tracking performance of postgraduate pediatric dental students in terms of detecting abnormalities correctly in different sets of panoramic radiographs. The research question is: Is there a significant difference in the ETT measures of postgraduate pediatric dental students using different sets of panoramic radiographs with abnormalities? The null hypothesis is that there are no differences in the eye-tracking measures between radiographs with AOIs located in one sextant and radiographs with AOIs located in multiple sextants. The alternative hypothesis is that there are differences in the eye-tracking measures between radiographs with AOIs located in one sextant and radiographs with AOIs located in multiple sextant(s).

## 2. Materials and Methods

### 2.1. Ethical Approval

The Ethics Committee at King Abdulaziz University Dental Hospital (KAUFD) approved this study (Approval number: 112-10-22, date: 15 November 2022). This study adhered to the guidelines outlined in Strengthening the Reporting of Observational Studies in Epidemiology (STROBE) [20]. Informed consent was obtained from all participants before commencing the computer-based tests.

### 2.2. Study Participants and Stimuli

In this cross-sectional study, we included all current pediatric postgraduate dental residents at King Abdulaziz University Dental Hospital (KAUFD), which is the major public dental school in Jeddah, Saudi Arabia, holding both the national and American Dental accreditation. One of the core courses taught to postgraduate pediatric dental residents at King Abdulaziz University is Oral and Maxillofacial Radiology, a one-credit course in the first year of residency. This course, including its teaching materials and exams, are comprehensively provided by the Oral and Maxillofacial Radiology department at the school of dentistry. Convenience sampling was used in this study. An invitation email was sent to all 32 pediatric dental residents at KAUDH to voluntarily participate in the study. Those who agreed to participate were included in the study after receiving an explanation of its objectives. To encourage a higher response rate, the participants were offered a store gift card as an incentive.

Undergraduate dental students and pediatric postgraduate dental students outside of KAUDH were excluded. Subjects who declined to participate in the study and those with a gaze deviation greater than or equal to ≥1° upon calibration were also excluded.

Seven panoramic radiographs (Appendix A) of pediatric dental patients aged between 6 and 16 years of age were selected from the electronic health system of KAUDH, based on their quality. Case selection mandated the presence of at least one bony lesion. A panel of experts from KAUDH, including three pediatric dentists and an oral radiologist, reviewed the selected cases to ensure that the radiographs were of good quality. All the images used in the study underwent no manipulation. The type of lesion was recorded for each patient and confirmed by their dental records. All identifying data were removed and a de-identified serial number was assigned to each case. The panoramic radiographs in this study presented a wide variety of abnormalities that included deviated nasal septum and enlarged nasal conchae; fibrous dysplasia; regional odontodysplasia or ghost teeth; dentigerous cysts; odontogenic keratocysts; enlarged dental follicle; missing, impacted, and rudimentary teeth; dilacerated roots; retained primary teeth; and periapical radiolucencies.

### 2.3. Eye-Tracking System and Measures

The study was conducted in a quiet room that with dim lighting and no distractions within the field of view. A 15.6-inch laptop screen (Latitude E6530, Dell Corporation, Round Rock, TX, USA) was used, and the eye tracker was securely positioned at the base of the screen using a magnetic strip. RED-m^®^ SMI software (Sensomotoric Instruments, Teltow, Germany) was used to track the participants’ eye movements as they viewed the radiographs displayed on the screen. Before the experiment, specific areas of interest (AOIs) were identified on each radiograph. To ensure consistency among the participants and to calibrate their eyes to the eye-tracker, the experiment began with an eye tracker calibration exercise. Calibration was performed to ensure accurate alignment of the eye movement pattern with the displayed images.

First, each participant received verbal instructions and a thorough explanation of the experiment (Appendix B). They were then instructed to sit comfortably, with the distance between the participant’s eyes and the screen ranging from 50 to 75 cm. Following calibration, the participants examined the radiographs and answered related questions. The test began with a demographic section, wherein participants provided information on their gender and academic level. Subsequently, panoramic radiographs were presented on the screen consistently for all participants. Each radiograph was examined individually, and the participants responded to questions regarding the presence and location of abnormalities. For each panoramic radiograph presented, the participants were asked three questions. First, they were asked whether there were any abnormalities in the presented radiograph. Second, they were asked to locate the abnormality by selecting one or more sextants with the panoramic radiograph or to choose that there was no abnormality present. Lastly, if there was an abnormality, they were asked to give a differential diagnosis or a brief description. The last question was just to ensure that the participants described the targeted abnormality. The questionnaire was developed based on the authors’ scientific backgrounds and a thorough literature review. To measure the test–retest reliability, we divided the test into three sections: lesion detection, lesion location questions, and a total score. For lesion detection, a score of 0 was given for “no abnormality” and a score of 1 for “yes, there is an abnormality”. For lesion location, the score ranged from 0 to 6, with 0 points for “no abnormality” and 1 point for each sextant where an abnormality was reported. The test–retest reliability was assessed with a two-week interval between the two measurements. Reliability was analyzed using Cronbach’s alpha for lesion detection, lesion location questions, and the total score. The data collected from each participant were imported automatically from the experimental software into the analysis software. The software also outlined AOIs corresponding to the identified abnormalities.

The data collected for each radiograph included revisit counts, fixation counts, fixation time, entry time, and dwell time. Revisit counts were defined as the number of times a participant’s gaze returned to a specific AOI on the radiograph after looking away. Fixation count was defined as the number of times a participant’s gaze was stationary on a specific AOI on a radiograph, whereas fixation time was the amount of time a participant’s gaze was stationary on a specific AOI on a radiograph, measured in milliseconds. The entry time was defined as the amount of time it took for a participant’s gaze to land on a specific AOI on a radiograph, measured in milliseconds. Finally, dwell time was the amount of time a participant spent looking at a specific AOI on a radiograph, and was measured in milliseconds.

### 2.4. Statistical Analyses

Descriptive analyses were conducted to summarize the participants’ characteristics and eye-tracking performances using eye-tracking measures and satisfaction bias. Data were presented as frequencies, percentages, medians, and the associated 25th and 75th interquartile ranges (IQR). Two of the cases had multiple AOIs located in multiple sextants. Successful detection of one AOI and missing the others in a single radiograph was considered satisfaction of search (SOS) bias [21]. 

Bivariate analyses were conducted to examine differences in participants’ performances as well as ET measures using different sets of panoramic radiographs. A two-sample test of proportions was conducted to determine whether pediatric postgraduates were able to correctly locate the AOIs in the two different groups of stimuli. Statistical significance was set at *p* < 0.05. Furthermore, the Mann–Whitney U test was used to test the equality of the eye-tracking measures for radiographs with AOIs located in a single sextant versus multiple sextants. We further performed a Bonferroni correction to account for multiple testing and reduce the chance of a type I error; therefore, the *p*-value was reduced to 0.01.

In addition, the cumulative duration for which the participants gazed at each AOI on each panoramic radiograph was calculated, resulting in heat maps (graphical color-coded maps). These heat maps represent a visualization technique that presented the distribution of eye fixation positions over time [22]. 

## 3. Results

Table 1 shows the characteristics of the panoramic radiographs used in this study. Seven radiographs with abnormalities were included in the eye-tracking test analysis. The number of AOIs in each radiograph ranged from one to eight. This generated 540 observations from 30 students. The radiographs were classified according to the number of sextants involved. Thus, they were divided into: (1) radiographs with AOIs located in a single sextant and (2) radiographs with AOIs located in multiple sextants.

Table 2 summarizes the participants’ characteristics. Most of the respondents were female (73.3%). Approximately 66.7% were between the ages of 26 and 30. Their years of clinical experience after graduation were 2–4 years for 56.7% of the participants (n = 17) and 5 or more years for the remaining participants (43.3%).

Overall, the percentage of correctly located AOIs was 71.7%. Correct diagnosis was reported in 71.0% of the radiographs with a single involved sextant compared to 72.1% of the radiographs with multiple involved sextant(s). The two-sample test of proportions indicated no statistically significant differences between the proportions of correctly located AOIs for both types of radiographs (single- or multiple-involved sextant(s).)

Regarding satisfaction search bias in this study, it was found in one of the two cases with multiple involved sextants, where 76.7% (n = 23) demonstrated SOS, and only 20% of the participants (n = 6) did not demonstrate any level of SOS. 

Table 3 summarizes the key ETT measures for each recorded AOI based on the number of sextants involved in the radiographs. Overall, the median scores for revisits and fixation counts for the AOIs were four [IQRs: 1–8 and 0–11, respectively]. In radiographs with AOIs located in one sextant, we found that residents had significantly more revisits (5 [IQR: 3–10]) and fixation counts (9 [IQR: 4–20]) than those in radiographs with AOIs located in multiple sextants (*p* < 0.001). Similar patterns were observed for fixation and dwell times (*p* < 0.001). In radiographs with AOIs located in multiple sextants, we found that residents had slower entry times and dwelled less on AOIs in radiographs with AOIs located in multiple sextants than on radiographs with AOIs located in one sextant. The abnormalities that were frequently missed by the participants were deviated nasal septum and enlarged nasal conchae, odontogenic keratocysts, and rudimentary teeth.

**Figure 1:**
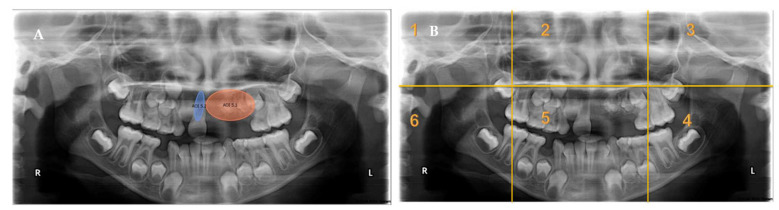

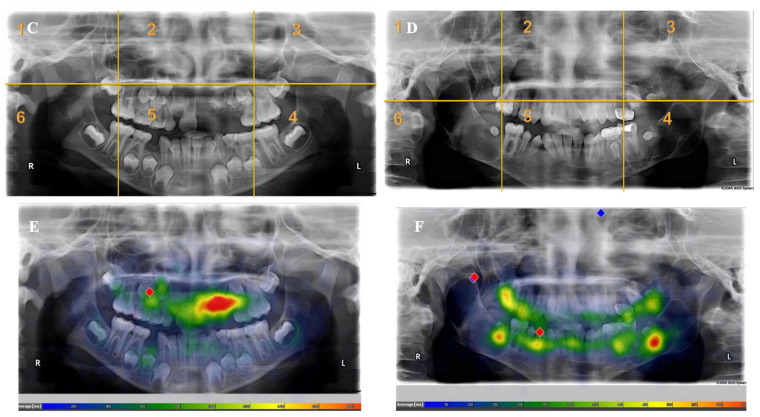
An example of a panoramic radiograph with areas of interest (AOIs) presented divided into sextants. (**A**) Panoramic radiograph with AOIs located in a single sextant presenting regional odontodysplasia, missing central incisor, and wide pulpal canal of tooth # 11. (**B**) Panoramic radiograph with AOIs located in multiple sextants presenting rudimentary second molars, odontogenic keratocyst, retained primary tooth, and missing lower permanent canine. (**C**) Divided sextants in a panoramic radiograph with AOIs located in a single sextant. (**D**) Divided sextants in a panoramic radiograph with AOIs located in multiple sextants. (**E**,**F**) Heatmap buildups from data registered for participants. (**E**) Postgraduate residents spent more time fixating on AOI located in one sextant than in other sextants. (**F**) Some AOIs had a considerably higher concentration of fixations than other AOIs. The colors of the heatmap range from a blue color, which indicates less fixation time, to a red color, which indicates a greater fixation time, all in milliseconds.

Figure 1 presents a heatmap of one single-involved-sextant radiograph and one multiple-involved-sextants radiograph. Heatmaps were generated to represent the areas with the longest fixations over time. The highest fixation density was on the AOIs, with minimal attention paid to the other areas of the panoramic radiograph. Residents fixated more on dentition than on bony structures.

## 4. Discussion

This study explored the eye-tracking performance of postgraduate pediatric dental residents in terms of correctly detecting abnormalities on different sets of panoramic radiographs. We found that over two-thirds of the AOIs were correctly detected by the pediatric dental residents, with slightly higher proportions in multiple-sextant radiographs. In single-sextant radiographs, residents had significantly more revisits and fixation counts than in radiographs with AOIs located in multiple sextants, and similar patterns were observed for fixation and dwell times. On radiographs with AOIs located on multiple sextants, residents dwelled less on the AOIs and had slower entry times than those with AOIs located on one sextant. The generated heatmaps showed that the residents fixated more on dentition than on the bony structures.

This study observed a slightly higher percentage of correctly detected abnormalities on multiple-lesion radiographs. Radiographs with more than one lesion tended to be seen among syndromic cases or were associated with other lesions [23]. We speculate that pediatric dental residents may have utilized their previous clinical knowledge of such phenomena, and the presence of one lesion sparked the search for another. Similar behavior was observed among neurologists when examining brain CT scans. The brief clinical introduction and diagnosis of the cases affected their scanning pattern, and they scanned areas where abnormalities might be found [24]. However, radiographs with single lesions might tend to be less diagnostically distinct, which increases the chance of missing them or their detection during routine screening. 

The overall high percentage of correct diagnoses (71%) among the participants was probably due to their advanced educational training, which taught pediatric radiographic evaluation alongside clinical presentation and management. Despite the relatively high percentage of correct diagnoses, SOS bias was recorded in both cases involving multiple sextants. This type of bias occurs when the clinician tends to miss a significant finding after detecting an abnormality in an image [25] due to a faulty visual search or recognition errors [26]. SOS can be overcome with a systematic scan pattern when evaluating dental radiographs. In dentistry, little attention has been given to evaluation of the possible existence of SOS bias during dental radiograph interpretations [27]. According to a previous study, a true SOS error occurred in 20% of the interpretations of periapical radiographs [27]. Future studies should evaluate the existence of SOS bias during panoramic radiograph interpretations in a larger group of dental images with multiple lesions to minimize false-negative errors.

In radiographs with AOIs located in one sextant, the postgraduate residents had significantly more revisits, fixation counts, fixation times, and dwell times than in radiographs with AOIs located in multiple sextants. Although the students focused more on the AOIs of the single-sextant radiographs, they presented faster entry times. On multiple-sextant radiographs, they were slower to locate the lesions. This could be due to the diversion of attention to various locations in multiple-involved-sextant radiographs, shortening their fixation and dwelling, but lengthening their entry time. 

Heatmapping using ETT provides a visual demonstration of cumulative fixation concentration over time, which is presented as a color in a predefined color scheme. The colors range from blue to red, where red indicates longer fixations [28]. In this study, the highest density of fixations was observed on the AOIs and dentition, with slight attention paid to the other areas of the panoramic radiograph. This was a double-edged sword, as the goal was to have postgraduate dental students locate and identify lesions when observing dental radiographs; however, a thorough examination of these radiographs is of equal importance.

Furthermore, we need to understand the clinicians’ scan patterns when viewing radiographs to suggest the best ways to overcome missing lesion identification [25]. Several suggestions have been proposed to minimize interpretational errors and enhance lesions’ detection, such as double reading and computer-aided detection [25]. To ensure comprehensive radiographic evaluation, an educational strategy of a standardized scan scheme of panoramic radiographs is suggested, which starts with screening the periphery (bone, temporomandibular joints, etc.) and moves to the central region [29]. Also, it is advantageous to divide each radiograph into sextant(s) and to give each sextant an equal amount of time, regardless of whether a lesion was found or not. This was suggested because the center of the panoramic radiograph attracts the most attention [5], and we intended to avoid focusing on the middle segment of the radiograph or missing the periphery. A recent study evaluated the eye movement pattern when observing panoramic radiographs of patients at their mixed dentition stages. They classified the eye movement patterns while reading panoramic radiographs into three categories: R (clockwise rotation), L (counterclockwise rotation), and S (mainly saccades). They recommended that observations be made in a clockwise pattern when interpreting panoramic radiographs during the mixed dentition phase, as it’s associated with enhanced learning performance. They concluded that frequent eye movements could signal reduced interest or challenges with the task, and that they are linked to inferior learning performance [30].

Numerous studies have been conducted on the use of ETT in different fields; however, integrating its use into education has gained attention only in the past few years [31]. Recently, it has been proposed that ETT can facilitate the development of virtual reality (VR) and augmented reality (AR) simulations for education and training [32]. By tracking where learners direct their gazes in these virtual environments, developers can ensure that essential details are not overlooked and create more immersive and realistic training scenarios. This technology can enhance the hands-on training experiences, allowing users to practice complex procedures and gain confidence in a safe and controlled environment. 

Moreover, ETT can act as an aid for students with special care needs [33]. For example, individuals with attention deficit hyperactivity disorder (ADHD) can greatly benefit from personalized interventions based on their eye tracking data [34]. By tracking their eye movements, educators can gain insight into their attention patterns, identify potential triggers for distraction, and develop targeted strategies to improve focus and concentration [35].

The findings of this study must be interpreted within its limitations. First, this study was conducted at a single center, which can limit its generalizability. However, King Abdulaziz University has one of the largest pediatric dental residencies in the country, and its accepted candidates have graduated from different dental schools in the country. Second, a limited number of radiographs was used in this study. However, the reason was to improve the response rate and response quality, as well as to prevent eye fatigue during the eye-tracking process. Another limitation of this study was the lack of a control group, which renders a comparison with more or less experienced participants not possible. Future studies should perform similar investigations on radiographs used in advanced clinical training, such as cone-beam computed tomography radiographs.

## 5. Conclusions

We found that over two-thirds of the AOIs were correctly detected by pediatric dental residents. In single-sextant radiographs, residents had significantly more revisits, fixation counts, and dwell time compared to radiographs with AOIs located in multiple sextants. In radiographs with AOIs located in multiple sextants, residents dwelled less on AOIs and had slower entry times. The generated heatmap showed that residents fixated more on the dentition compared to the bony structures. The reported findings can help us to develop a standardized scan scheme of panoramic radiographs that can be taught to dental students in their dental programs to improve their performances and minimize recognition errors.

## Figures and Tables

**Table 1:** Panoramic radiograph characteristics.

Radiographs with Abnormalities	Number of Sextants Involved	Number of AOI ^a^	Number of Participants	Total Recorded Observation ^b^
Radiograph 1	Multiple	3	30	90
Radiograph 2	Multiple	8	30	240
Radiograph 3	Single	2	30	60
Radiograph 4	Single	2	30	60
Radiograph 5	Single	1	30	30
Radiograph 6	Single	1	30	30
Radiograph 7	Single	1	30	30
Overall Observations				540

^a^ **AOI**: area of interest. ^b^ **Total recorded observation**: number of participants multiplied by the AOI.

**Table 2:** Participants’ characteristics.

Participants’ Characteristics	n (%)
	Pediatric Postgraduate
	Dental Students
**Overall**	30 (100)
**Sex**	
Female	22 (73.3)
Male	8 (26.7)
**Age Categories (Years)**	
20–25	0 (0.0)
26–30	20 (66.7)
31–35	7 (23.3)
36–40	3 (10.0)
**Clinical Experience (Including Internship Year)**	
1 year or less	0 (0.0)
2–4 years	17 (56.7)
5 or more years	13 (43.3)

**Table 3:** Eye tracking key measures for pediatric postgraduate dental students for each recorded AOI based on the number of sextants involved in the radiographs.

Eye Tracking Measures	Median Score (25th–75th IQR)			Mann–Whitney U Test Test Statistic [*p*-Value *]
	Overall	AOIs Located in One Sextant	AOIs Located in Multiple Sextants	
**Revisit Count** **(Frequency Count)**	4 (1–8)	**5 (3–10)**	**2 (0.5–5)**	7.338 [<0.001]
**Fixation Count** **(Frequency Count)**	4 (0–11)	**9 (4–20)**	**1 (0–6)**	10.273 [<0.001]
**Entry Time (Milliseconds)**	3551.9 (1073.3–11,721.4)	2878 (941.4–11,062.7)	4000.1 (1227–13,907.25)	−1.918 [0.055]
**Fixation Time (Milliseconds)**	1389.15 (0–3591.9)	**2736.8** **(1335–6040.6)**	**550.7** **(0–2269.3)**	9.740 [<0.001]
**Dwell Time (Milliseconds)**	1414.15 (0–3696.35)	**2820.35** **(1335–6207.5)**	**550.7** **(0–2286.1)**	9.782 [<0.001]

**IQR:** interquartile range. **AOI:** area of interest. Bold font indicates statistical significance. (*): Alpha level is 0.01 after Bonferroni correction.

## Data Availability

The data created and analyzed during this study are available upon request from the corresponding author.

## Appendix B. Instructions Prior to Commencing the Questionnaire

The eye-tracking technology is a device for measuring eye positions and eye movement.

- The test will take around 15 to 20 min to be completed.
- You will start with a calibration process that takes less than a minute.
- Each panoramic radiograph displayed will be divided into sextants by yellow lines.
- The test contains seven cases, with three questions related to each case.
- Feel free to move to the next slide once the radiograph has been examined.
- This is a one-time test, and you cannot move backward on the slides.
- If you are wearing eyeglasses or lenses, or have had any kind of eye operation, please write them in the description box before starting the test.
- Clinical experience includes the internship year.
- You can press (F12) on the keyboard to exit the exam.
- Please do not share the questions after completing the test.
